# From macrofungal polysaccharides to precision microbial immunotherapy: reprogramming the tumor microenvironment through immunometabolic and gene-regulatory pathways

**DOI:** 10.3389/fonc.2026.1966586

**Published:** 2026-10-12

**Authors:** Kingsley Excel Dunga, Sylvester Chibueze Izah, Ebinyo Rebecca Aseibai, Matthew Chidozie Ogwu, Vivian Ibienebakabobo Promise, Nsikak Godwin Etim, Irene Nneka Ohanusi

**Affiliations:** 1 Department of Medical Laboratory Science, Kingsley Ozumba Mbadiwe University, Ogboko, Ideato, Imo, Nigeria; 2 Department of Community Medicine, Faculty of Clinical Sciences, Bayelsa Medical University, Yenagoa, Bayelsa, Nigeria; 3 Department of Microbiology, Faculty of Science, Bayelsa Medical University, Yenagoa, Bayelsa, Nigeria; 4 Department of Microbiology, Federal University Otuoke, Otuoke, Bayelsa, Nigeria; 5 Goodnight Family Department of Sustainable Development, Appalachian State University, Boone, NC, United States; 6 Department of Public Health, Faculty of Health Sciences, Bayelsa Medical University, Yenagoa, Bayelsa, Nigeria; 7 Niger-Delta Institute for Emerging and Re-emerging Infectious Diseases, Federal University Otuoke, Yenagoa, Bayelsa, Nigeria; 8 Department of Microbiology, Faculty of Natural Sciences, Hezekiah University, Umudi, Nkwerre, Imo, Nigeria

**Keywords:** Dectin-1, immunometabolism, macrofungi, microbial immunotherapy, synthetic biology, trained immunity, tumor microenvironment, β-glucan

## Abstract

Engineered microbial immunotherapies can localize immune-modulating signals within solid tumors, where hypoxia, suppressive myeloid cells, deficient antigen presentation, and dysfunctional T-cell trafficking often limit immunotherapy. Macrofungal polysaccharides, especially structurally defined β-glucans, offer mechanistic templates rather than interchangeable drug substances for source-specific, structure-controlled immune engineering programs. Their activity depends on linkage, branching, molecular mass, conformation, solubility, aggregation, and physical presentation. Critically, the strongest Dectin-1 and trained-immunity evidence discussed here uses particulate β-glucans from Saccharomyces cerevisiae; those findings cannot be assigned directly to macrofungal β-glucans without material-specific comparison. We therefore distinguish four engineering architectures: *de novo* microbial biosynthesis of a defined β-glucan, microbial surface display, delivery of a pre-characterized β-glucan cargo, and delivery of non-glycan payloads that mimic the desired downstream immune program. Because therapeutic bacteria have not yet been shown to reproduce a macrofungal β-glucan with controlled linkage, branching, conformation, and presentation, near-term development should prioritize standardized cargo delivery or pathway mimicry. In pancreatic and colorectal cancer models, yeast whole-glucan particles have altered myeloid trafficking or generated epigenetically and metabolically trained macrophages linked to CXCL9/CXCL10 production, natural killer-cell recruitment, conventional type 1 dendritic-cell accumulation, and stronger CD8 T-cell responses. Human evidence remains preliminary and cannot establish β-glucan-specific efficacy in uncontrolled combination studies. This Perspective proposes source-resolved material characterization, receptor and cell-state testing, multidimensional pharmacodynamic biomarkers, and microbial safety controls as prerequisites for translating fungal-inspired immune programs into precision cancer therapeutics.

## 1 Introduction

The immunosuppressive tumor microenvironment (TME) is a major barrier to effective treatment of many solid malignancies. Tumor-associated macrophages, suppressive myeloid populations, dysfunctional antigen-presenting cells, altered nutrient availability, hypoxia, and inhibitory cytokine networks can restrict T-cell entry and function even when an immune checkpoint is therapeutically blocked. Engineered bacteria offer a complementary strategy because selected microbial chassis can be programmed to localize immune-active payloads within tumors rather than expose the whole body to the same signal. Preclinical platforms have used synchronized bacterial lysis, local checkpoint-blockade nanobodies, metabolic rewiring, and innate immune agonists to reshape the TME (1–4). The first-in-human SYNB1891 study further showed that an engineered Escherichia coli Nissle strain can engage the intratumoral stimulator of interferon genes (STING) pathway and generate tissue-level pharmacodynamic signals (5).

The next challenge is therefore not only whether microbes can be engineered, but which biological programs should be engineered into them. Macrofungi offer an underused source of such programs. Edible and medicinal mushrooms contain β-glucans, other polysaccharides, proteins, terpenoids, and phenolic compounds with reported immunomodulatory activity (6, 7). Recent studies have highlighted the immune-supporting potential of edible-mushroom bioactives and the preclinical anticancer relevance of mushroom-derived compounds, while emphasizing the need for stronger mechanistic and clinical validation (8, 9). These contributions are useful as a biological starting point, not as proof of clinical efficacy.

This Perspective advances a narrower proposition: structurally defined macrofungal polysaccharides should be treated as immune-programming templates for microbial oncology. Four distinct implementation routes must be evaluated rather than conflated: *de novo* microbial biosynthesis, microbial surface display, delivery of a pre-characterized β-glucan cargo, and delivery of a non-glycan payload that mimics the desired downstream immune state. The near-term emphasis is on the last two routes because none of the cited studies demonstrates that a therapeutic bacterial chassis can reproducibly build or display a macrofungal β-glucan with controlled linkage, branching, conformation, and particle geometry.

## 2 From mushroom bioactivity to mechanistically defined design rules

“Mushroom polysaccharide” is chemically too broad to function as a therapeutic mechanism. β-Glucan activity varies with glycosidic linkage, branching, molecular mass, tertiary conformation, solubility, aggregation, and particulate presentation (10–13). These properties influence receptor accessibility and intracellular handling; therefore, two materials described as β-glucan can generate different immune outputs. Macrofungal products also differ from yeast-derived whole-glucan particles, and results from one preparation should not be generalized automatically to another.

This variability matters because Dectin-1 signaling depends partly on how β-glucan is physically presented. Horneck Johnston et al. (14) used particulate whole-glucan particles derived from Saccharomyces cerevisiae and showed that Dectin-1 phagocytic-synapse formation and lysosomal signaling were associated with the metabolic reprogramming required for trained immunity. This is yeast, not macrofungal, evidence. It is therefore a mechanistic comparator rather than proof that mushroom-derived β-glucans will generate the same response. Goodridge et al. (15) established Dectin-1 as a major innate receptor for fungal β-glucans. while later structure-function syntheses reinforced the importance of molecular organization (10, 11).

Evidence is therefore classified by source and physical form. Macrofungal evidence includes lentinan and heterogeneous mushroom preparations; yeast evidence includes purified S. cerevisiae whole-glucan particles with a particulate β-(1→3)-glucan-rich wall. A shared receptor label does not demonstrate pharmacological equivalence. Direct transfer requires compositional analysis, matched-dose and matched-presentation assays, Dectin-1-dependence testing, and confirmation that both materials generate the same metabolic, epigenetic, and cellular-state outputs.

The clinical literature also argues against treating mushrooms as a single therapeutic class. Narayanan et al. (16) screened 2,349 records, identified 136 clinical studies for detailed consideration, and included 39 studies covering 12 mushroom preparations. Eleven included studies reported favorable immunological responses and 14 reported improvements in quality of life or symptom burden, but study designs, preparations, cancer types, concomitant treatments, and endpoints were heterogeneous. The review therefore concluded that evidence remained insufficient to recommend routine mushroom supplementation in cancer care. This distinction is essential: a heterogeneous natural-product literature can motivate mechanistic research without establishing therapeutic efficacy.

Older clinical evidence with lentinan illustrates both promise and uncertainty. In an individual-patient-data meta-analysis of five gastric-cancer trials comprising 650 patients, lentinan combined with chemotherapy was associated with longer overall survival than chemotherapy alone (hazard ratio [HR] = 0.80, 95% confidence interval [CI] 0.68–0.95; p = .011) (17). However, formulation, treatment era, population, and co-therapy limit transferability to modern immunotherapy. The appropriate lesson is that defined fungal glycans warrant receptor-resolved, biomarker-led study—not that any β-glucan is a proven cancer immunotherapy. **Table 1** summarizes the current evidentiary bridge, and **Table 2** makes source, formulation, and transferability explicit.

**Table 1:** Quantitative evidence informing a macrofungal-to-engineered microbial immunotherapy framework.

References	Model/intervention	Quantitative or mechanistic finding	Interpretation for this perspective
Narayanan et al. (16)	Systematic review of human mushroom studies in cancer	2,349 records screened; 39 studies included; 12 preparations represented; 11 studies reported favorable immunological responses and 14 reported quality-of-life/symptom benefits	Human evidence exists but is heterogeneous and insufficient for routine clinical recommendation; mechanism-specific development is needed
Oba et al. (17)	Individual-patient-data meta-analysis of lentinan plus chemotherapy in advanced gastric cancer	650 patients from 5 trials; overall survival HR = 0.80 (95% CI 0.68–0.95), p = .011	Provides a historical clinical signal for a defined fungal β-glucan, but does not establish generalizability to modern immunotherapy
Geller et al. (18)	Yeast whole-glucan-particle training in pancreatic cancer models	Dectin-1 deletion reduced pancreatic β-glucan trafficking by about 5-fold; pancreatic CD45+ immune cells increased about 7-fold at day 7; CCL2 increased about 30-fold at 24 h	Demonstrates receptor-dependent trafficking and a measurable myeloid-reprogramming cascade relevant to TME design
Chu et al. (19)	Phase Ib, single-arm WGP β-glucan + camrelizumab + S-1/oxaliplatin (SOX) in advanced gastric adenocarcinoma; β-glucan source/lot structure not fully reported	n = 30; objective response rate (ORR) 60% (95% CI 41.4–78.6); disease-control rate 93.3% (95% CI 77.9–99.2); median progression-free survival (PFS) 10.4 months (95% CI 9.52–11.27); median overall survival (OS) 14.0 months (95% CI 11.09–16.91); grade ≥3 treatment-related adverse events 30%	Suggestive combination activity, but the single-arm design and three-component regimen prevent attribution of effect to β-glucan
Luke et al. (5)	Phase I intratumoral SYNB1891, with/without atezolizumab	32 treated (24 monotherapy, 8 combination); 9/25 evaluable participants had stable disease; no SYNB1891-related infections; intratumoral interferon/STING-response genes increased	Shows that engineered bacteria can deliver a defined immune program and generate human pharmacodynamic readouts

CI, confidence interval; HR, hazard ratio; ORR, objective response rate; OS, overall survival; PD-1, programmed cell death protein 1; PD-L1, programmed death-ligand 1; PFS, progression-free survival; SOX, S-1 plus oxaliplatin; STING, stimulator of interferon genes; WGP, whole-glucanparticle.

**Table 2:** Source, physical form, quantitative result, and transferability boundary.

References	Material/provenance	Verified quantitative result	Boundary for interpretation
Oba et al. (17)	Lentinan, a macrofungal β-glucan preparation, plus chemotherapy	Individual-patient-data meta-analysis: 5 trials, n = 650; OS HR = 0.80 (95% CI 0.68–0.95), p = .011	Historical combination signal; not evidence that yeast particles or engineered-microbial products are equivalent
Geller et al. (18)	Purified particulate WGP from S. cerevisiae; reported particle diameter 2–4 μm	Dectin-1 deletion: ~5-fold lower pancreatic trafficking; pancreatic CD45+ cells: ~7-fold higher at day 7; CCL2: ~30-fold higher at 24 h	Mechanistic pancreatic-cancer evidence for yeast WGP, not macrofungal β-glucan
Horneck Johnston et al. (14)	Nonsoluble, nonaggregated yeast WGP from S. cerevisiae	Training experiments tested 1, 10, and 100 μg/mL yeast WGP; biological activity varied with particle recognition and signaling context	Shows form- and Dectin-1-dependent yeast-particle biology; transfer needs head-to-head material testing
Wang et al. (20)	Oral yeast-derived WGP with PD-1/PD-L1 blockade in heterogeneous advanced cancers	Exploratory cohort n = 13; disease-control rate 69.2%; median PFS 3.67 months; median OS 8.0 months	Small, uncontrolled combination cohort; efficacy cannot be attributed to WGP
Chu et al. (19)	Oral WGP β-glucan + camrelizumab + SOX; source and structural attributes not fully specified in the report	Phase Ib, n = 30; ORR 60% (95% CI 41.4–78.6); median PFS 10.4 months; median OS 14.0 months; grade ≥3 treatment-related adverse events 30%	Single-arm, three-component regimen prevents β-glucan-specific attribution
Hamdan et al. (21)	Whole-glucan particles derived from yeast cell walls in CT26 colorectal-cancer experiments	Tumor-growth experiments used n = 8 mice/group; macrophage-infiltration analyses used n = 6/group	Preclinical pathway evidence for yeast particles; macrofungal transfer requires structural and functional equivalence

CI, confidence interval; HR, hazard ratio; ORR, objective response rate; OS, overall survival; PD-1, programmed cell death protein 1; PD-L1, programmed death-ligand 1; PFS, progression-free survival; SOX, S-1 plus oxaliplatin; WGP, whole-glucan particle.

## 3 Dectin-1 is a context-dependent control point, not a universal “on” switch

Dectin-1, encoded by CLEC7A, is among the most studied receptors for fungal β-glucans, but its role in cancer is not uniformly antitumor. Wattenberg et al. (22) showed that simultaneous activation of Dectin-1 and CD40 generated potent antitumor myeloid activation in preclinical models, including checkpoint-resistant pancreatic tumors, and depended on conventional type 1 dendritic cells (cDC1s) and T-cell responses. Such findings suggest that fungal-glycan sensing can become therapeutically productive when embedded in the appropriate co-stimulatory context.

The opposite pattern has also been observed. Liu et al. (23) reported that Dectin-1-positive tumor-associated macrophages contributed to an immunosuppressive state in gastric cancer and that Dectin-1 blockade improved programmed cell death protein 1 (PD-1) inhibition in experimental models. In pancreatic cancer, Daley et al. (24) showed that Dectin-1 activation by galectin-9 promoted tolerogenic macrophage programming and adaptive immune suppression. These findings make a general “activate Dectin-1” rule biologically unsafe: ligand, receptor density, cell state, co-receptor signaling, tumor lineage, and spatial context can determine whether the receptor supports immunity or tolerance.

For a macrofungal-inspired therapy, CLEC7A expression alone should not be treated as a sufficient biomarker. Single-cell transcriptomics and spatial profiling should identify Dectin-1-positive myeloid states and connect them to phosphorylated spleen tyrosine kinase (Syk), antigen-presentation programs, chemokine output, and nearby T-cell function. A tumor-localizing chassis could then restrict exposure and pair a fungal-inspired stimulus with a second signal, such as CD40 co-activation, when the local state indicates that co-stimulation is required.

## 4 Trained immunity provides the gene-regulatory bridge

Trained immunity is the strongest gene-regulatory bridge between fungal sensing and tumor immunity. It describes a durable change in innate-cell responsiveness produced by metabolic and epigenetic reprogramming rather than antigen-specific lymphocyte memory (25). β-Glucan is a prototypical training stimulus. Cheng et al. (26) showed that protein kinase B (Akt), mechanistic target of rapamycin (mTOR), and hypoxia-inducible factor 1α (HIF-1α)-driven aerobic glycolysis are required for β-glucan-induced trained immunity. Saeed et al. (27) further showed persistent epigenetic reprogramming in trained monocytes, supporting the principle that a transient microbial signal can create a durable gene-regulatory state.

Cancer models extend that principle into the TME but do not erase material provenance. Kalafati et al. (28) showed that β-glucan training rewired granulopoiesis and generated neutrophils with antitumor properties. Geller et al. (18) specifically used purified, particulate whole-glucan particles from S. cerevisiae, not a macrofungal β-glucan. Dectin-1 deletion reduced pancreatic particle trafficking approximately fivefold; pancreatic CD45-positive immune cells increased approximately sevenfold at day 7, and pancreatic CCL2 increased approximately 30-fold at 24 hours. These quantitative intermediates connect exposure to myeloid trafficking and tumor control, but their transfer to macrofungal material requires direct testing.

Hamdan et al. (21) likewise used whole-glucan particles derived from yeast cell walls, not a macrofungal preparation. In colorectal cancer models, training enhanced vaccine activity through macrophage epigenetic and metabolic rewiring, including trimethylation of histone H3 lysine 4 (H3K4me3)-associated changes. Trained macrophages increased CXCL9 and CXCL10, promoting CXCR3-dependent natural killer (NK)-cell recruitment; NK-derived CCL5 supported cDC1 accumulation and stronger antigen presentation and effector-memory CD8 T-cell responses. Human peripheral-blood cells and patient-derived colorectal-cancer organoids supported elements of this pathway. The result defines a testable immune state, but not a class effect for mushroom β-glucans.

This precision also exposes safety questions. Trained immunity can persist, and durable inflammation is not universally beneficial. A microbial circuit should therefore be dose-controlled and spatially restricted, with blood and tissue pharmacodynamic markers that demonstrate the intended state without sustained systemic activation. Reversible or environmentally responsive circuits should be tested against continuous-expression designs rather than assuming that maximal innate activation is optimal.

## 5 From fungal biology to engineered living therapeutics

The feasibility of living delivery systems is supported by several independent microbial-oncology platforms, although none establishes microbial synthesis or display of a defined macrofungal β-glucan. Din et al. (3) engineered synchronized bacterial lysis to release genetically encoded cargo within tumors. Chowdhury et al. (2) and Gurbatri et al. (4) used programmable bacteria for local nanobody delivery, while Canale et al. (1) reprogrammed bacterial metabolism to increase intratumoral L-arginine. More recent systems have paired programmable bacteria with PD-1 blockade or dual immunostimulatory payloads (29, 30). These studies validate localization and payload control, not glycan equivalence.

SYNB1891 provides the clearest human proof of concept for mechanistically measured bacterial immunotherapy. In the phase I study, 24 participants received monotherapy and eight received combination treatment with atezolizumab. Among 25 evaluable participants, nine (36%) had stable disease, including four with stability beyond two months. Five cytokine-release-syndrome events occurred with monotherapy, including one grade 3 dose-limiting event; no SYNB1891-related infections were reported. Paired tumor biopsies showed induction of interferon-stimulated, chemokine/cytokine, and T-cell-response genes (5). This is evidence of target engagement and clinical feasibility, not β-glucan-specific efficacy.

Macrofungal biology could enter this engineering space through four technically distinct routes. First, *de novo* biosynthesis would require a chassis with validated glycosyltransferase or synthase activity, precursor supply, branch control, export, and release; product testing would have to confirm linkage, branch frequency, molecular-mass distribution, conformation, purity, and lot-to-lot potency. No cited therapeutic-bacteria study has achieved that specification for a macrofungal β-glucan. Second, surface display would add density, orientation, accessibility, and stability as critical attributes because receptor clustering depends on presentation. Display should therefore be treated as a separate, currently unvalidated architecture rather than as a synonym for production.

Third, a chassis could carry and release a pre-characterized β-glucan manufactured separately under defined quality controls. This route avoids asking the bacterium to synthesize the polymer, but loading, retention, release, structural integrity, particle size, and potency must be measured after formulation. Existing bacterial-lysis studies establish controlled release of genetically encoded payloads, not the delivery of a structurally validated β-glucan cargo; direct formulation and biodistribution studies are still required.

Fourth, a pathway-mimetic circuit could release non-glycan agonists, cytokines, chemokines, or metabolic payloads selected to reproduce a validated immune state without reproducing the polysaccharide. Near-term development should prioritize this route and pre-characterized cargo delivery. Success should be defined prospectively by receptor-proximal signaling, myeloid chromatin and metabolic markers, CXCL9/CXCL10 output, cDC1 and NK-cell recruitment, antigen presentation, and CD8 T-cell function. **Figure 1** summarizes this mechanism-led progression; the engineered endpoint remains a translational hypothesis.

**Figure 1:**
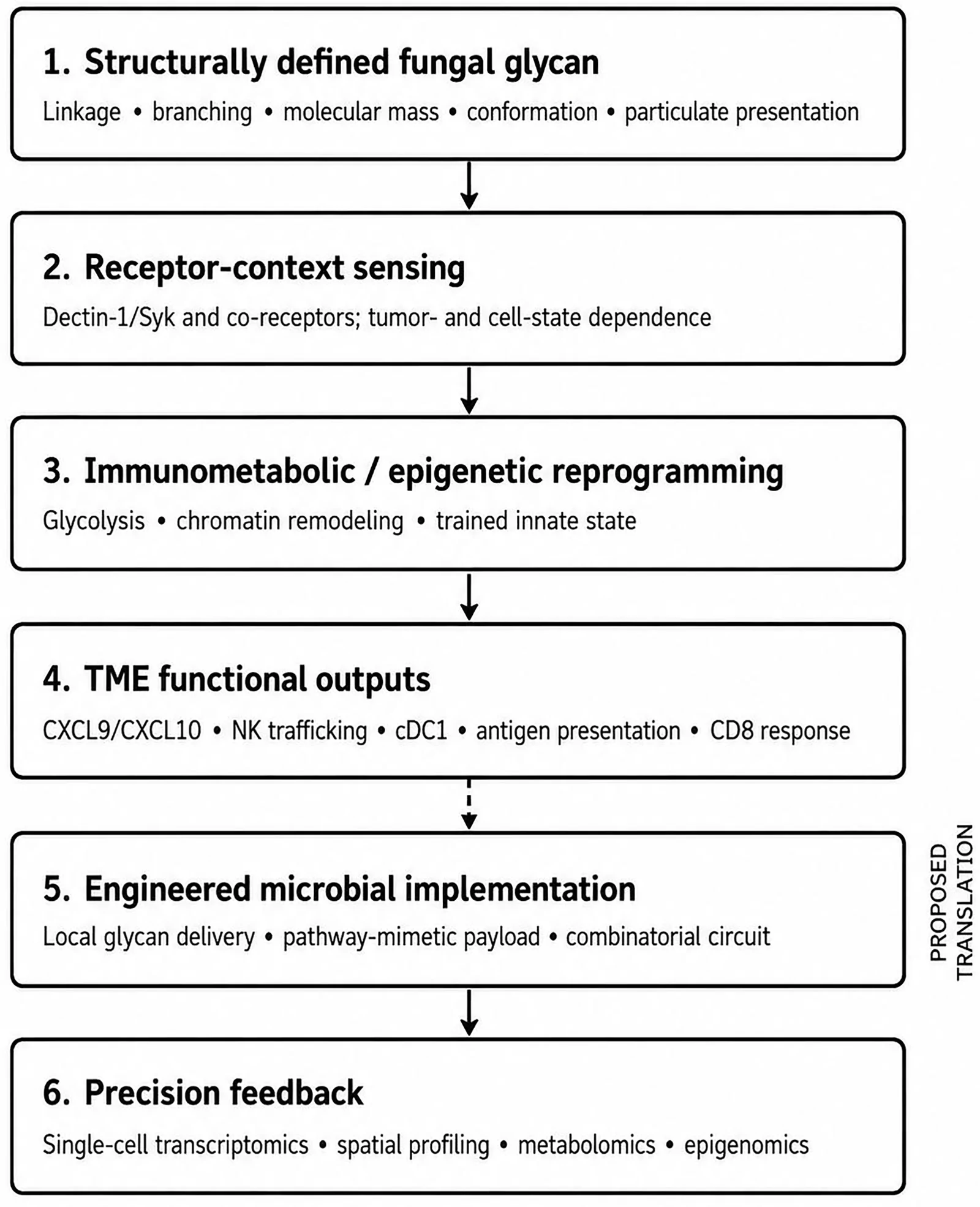
Proposed macrofungal-to-microbial immunotherapy framework. A mechanism-led framework linking structurally defined fungal polysaccharides to precision microbial immunotherapy. Evidence supports the influence of β-glucan structure and presentation on receptor sensing (14, 15), coupling of innate sensing to metabolic and epigenetic reprogramming (25, 26), and downstream myeloid/TME changes in cancer models (18, 21, 28). The final step—localizing a pre-characterized cargo or reproducing a fungal-inspired program with engineered microbial circuits—is a development hypothesis informed by current living-therapeutic platforms (1, 5, 31), not an established clinical pathway.

## 6 A translational agenda for precision development

A credible development pathway should begin with molecular definition. Candidate macrofungal polysaccharides should be characterized for linkage pattern, branch frequency, molecular-mass distribution, purity, conformation, solubility, aggregation, endotoxin, and batch consistency. Extract-level observations should be deconvoluted into structure-resolved fractions before biological comparisons. Chemically diverse preparations that share a common label may differ substantially in activity (6, 8, 9).

Second, receptor dependence and cellular state should be demonstrated rather than inferred. Genetic knockout, receptor-blocking, and signaling-inhibition experiments can separate Dectin-1-dependent effects from complement-receptor, Toll-like-receptor, or contaminant-associated responses. Head-to-head testing should compare the candidate macrofungal material with the exact yeast whole-glucan particle used as the mechanistic benchmark at matched mass, particle number, and accessible β-(1→3)/(1→6)-glucan content. Single-cell and spatial methods should then determine whether the responding state is associated with tumor control or immunosuppression.

Third, immunometabolism and epigenetics should become core pharmacodynamic endpoints. Metabolomics, extracellular-flux measurements, chromatin accessibility, and histone-mark profiling can establish whether acute receptor engagement produces a reproducible trained state. Candidate markers include H3K4me3-associated programs, glycolytic signatures, CXCL9/CXCL10 output, cDC1 accumulation, and spatial recruitment of effector lymphocytes (21, 26).

Fourth, engineering should follow the required biology. A particulate ligand requiring a phagocytic synapse needs a different architecture from a soluble agonist. A program that is antitumor only with CD40 signaling or checkpoint blockade should be built and evaluated as a controlled combination. Paired biopsies should distinguish inadequate microbial localization, insufficient payload, absent receptor competence, an unfavorable myeloid state, and downstream adaptive resistance; each failure mode implies a different redesign.

Clinical translation also requires a prospective safety package. The chassis should have characterized attenuation, genetic stability, antibiotic susceptibility, and a validated kill or clearance mechanism. Studies should quantify systemic dissemination, persistence in tumor and normal tissues, fecal or environmental shedding, and horizontal-transfer potential. Manufacturing controls must link identity, viability, payload output, structural attributes, release kinetics, purity, and potency to predefined specifications. For a β-glucan cargo, the critical quality attributes include linkage, branching, molecular mass, conformation, particle distribution, and endotoxin burden.

Dose escalation should incorporate stopping rules for cytokine-release syndrome, bacteremia or other infection, organ inflammation, and persistent systemic trained-immunity signals. Eligibility and monitoring should address severe immunosuppression, neutropenia, mucosal-barrier injury, implanted devices, and concurrent antibiotics because these factors may alter risk or exposure. SYNB1891 supplies useful precedent—one grade 3 dose-limiting cytokine-release event and no product-related infections were reported—but does not remove platform-specific risk (5). Go/no-go criteria should require tumor localization and intended tissue pharmacodynamics without sustained bloodstream detection, uncontrolled shedding, or systemic inflammatory activation.

## 7 Conclusion

Macrofungal polysaccharides should not be promoted as a shortcut from mushrooms to cancer immunotherapy. Their defensible value is as a library of immune-interacting structures from which transferable biological rules can be extracted. β-Glucan activity is conditioned by structure and presentation; Dectin-1 can support antitumor or tolerogenic programs depending on context; and trained immunity can connect fungal sensing to durable metabolic, epigenetic, and myeloid-state changes. However, much of the causal Dectin-1 and trained-immunity evidence summarized here comes from yeast whole-glucan particles. Human mushroom studies and early β-glucan combinations remain heterogeneous and do not establish a class effect.

Engineered microbial therapeutics could make this biology more precise, but the implementation route must be explicit. Current evidence most directly supports local delivery of a pre-characterized cargo or pathway-mimetic payload; *de novo* synthesis and surface display remain longer-term hypotheses requiring structural and functional validation. Progress should be judged across molecular structure, receptor engagement, signaling, metabolism, chromatin, cell state, spatial immune organization, safety, and clinical response. A future macrofungal-inspired microbial therapy may contain no mushroom-derived molecule; its connection to fungi may instead be the validated immune program it reproduces.

## Data Availability

The original contributions presented in the study are included in the article/supplementary material. Further inquiries can be directed to the corresponding author.
